# Urinary Retinol-Binding Protein: Relationship to Renal Function and Cardiovascular Risk Factors in Chronic Kidney Disease

**DOI:** 10.1371/journal.pone.0162782

**Published:** 2016-09-21

**Authors:** Maria Alice Muniz Domingos, Silvia Regina Moreira, Luz Gomez, Alessandra Goulart, Paulo Andrade Lotufo, Isabela Benseñor, Silvia Titan

**Affiliations:** 1 Nephrology Division, Department of Clinical Medicine, Faculty of Medicine, University of São Paulo, São Paulo, Brazil; 2 Nephrology Division, Kidney and Hypertension Hospital, São Paulo Federal University, São Paulo, Brazil; 3 Genetics Cardiovascular Laboratory, Heart’s Institute, Faculty of Medicine, University of São Paulo, São Paulo, Brazil; 4 Clinical Center Research, University Hospital, Faculty of Medicine, University of São Paulo, São Paulo, Brazil; University of Glasgow, UNITED KINGDOM

## Abstract

The role of urinary retinol-binding protein (RBP) as a biomarker of CKD in proximal tubular diseases, glomerulopathies and in transplantation is well established. However, whether urinary RBP is also a biomarker of renal damage and CKD progression in general CKD is not known. In this study, we evaluated the association of urinary RBP with renal function and cardiovascular risk factors in the baseline data of the Progredir Study, a CKD cohort in Sao Paulo, Brazil, comprising 454 participants with stages 3 and 4 CKD. In univariate analysis, urinary RBP was inversely related to estimated glomerular filtration rate (CKD-EPI eGFR) and several cardiovascular risk factors. After adjustments, however, only CKD-EPI eGFR, albuminuria, systolic blood pressure, anemia, acidosis, and left atrium diameter remained significantly related to urinary RBP. The inverse relationship of eGFR to urinary RBP (β-0.02 ± 95CI -0.02; -0.01, p<0.0001 for adjusted model) remained in all strata of albuminuria, even after adjustments: in normoalbuminuria (β-0.008 ± 95CI (-0.02; -0.001, p = 0.03), in microalbuminuria (β-0.02 ± 95CI (-0.03; -0.02, p<0,0001) and in macroalbuminuria (β-0.02 ± 95CI (-0.03; -0.01, p<0,0001). Lastly, urinary RBP was able to significantly increase the accuracy of a logistic regression model (adjusted for sex, age, SBP, diabetes and albuminuria) in diagnosing eGFR<35 ml/min/1.73m^2^ (AUC 0,77, 95%CI 0,72–0,81 versus AUC 0,71, 95%CI 0,65–0,75, respectively; p = 0,05). Our results suggest that urinary RBP is significantly associated to renal function in CKD in general, a finding that expands the interest in this biomarker beyond the context of proximal tubulopathies, glomerulopathies or transplantation. Urinary RBP should be further explored as a predictive marker of CKD progression.

## Introduction

Retinol binding protein (RBP) is a low molecular weight protein belonging to the lipocalin super family [1] and mainly synthesized in the liver. Its main function is to transport retinol (vitamin A). The majority of RBP-retinol circulates in the plasma bound to transthyretin (TTR) [2], a complex that prevents its glomerular filtration. Conversely, 4–5% of serum RBP-retinol circulates freely, pass the glomerular barrier and is then reabsorbed and degraded in the proximal tubule, a process mediated by megalin [3]. Due to these properties, urinary RBP is an established biomarker of proximal tubular dysfunction and is used as a diagnostic tool in proximal tubulopathies, such as Fanconi syndrome, Dent’s disease, cystinosis, or cast nephropathy.

Beyond the scenario of typical proximal tubulopathies, it has been shown that urinary RBP is related to the risk of CKD progression in some other conditions. Studies from one center have shown that higher values of urinary RBP are associated to disease activity, corticosteroid resistance and risk of CKD in diverse glomerulopathies [4,5]. In renal transplant recipients, urinary RBP has been positively associated to the risk of CKD [6], and in heart transplantation it has been associated with the risk of cyclosporine toxicity and CKD [7]. However, despite these promising initial results, urinary RBP has not been assessed in more prevalent CKD etiologies or in larger samples of CKD patients. In diabetic kidney disease, one of the most common causes of CKD worldwide, data are scarce, with few small cross-sectional studies in the 80´s showing that urinary RBP was associated to surrogate markers of progression such as microalbuminuria, HbA1c, and other parameters of microvascular disease [8–10].

Only recently, our group showed that urinary RBP was independently related to the risk of creatinine duplication or renal replacement therapy (RRT) initiation in macroalbuminuric diabetic kidney disease [11]. Along with previous findings in the literature, this result led us to the question whether urinary RBP could also work as a biomarker of CKD in conditions other than proximal tubulopathies, glomerulopathies and transplantation.

In this sense, the aim of the present study was to test the hypothesis that urinary RBP is associated with renal function in general CKD. For this we have evaluated the relationship between urinary RBP and renal function and cardiovascular risk factors in the baseline data of the Progredir Cohort Study, which comprehends stages 3 and 4 CKD participants. This cohort was designed to reflect more prevalent causes of CKD, by excluding primary and secondary glomerulopathies in enrollment, as well as renal transplant patients.

## Subjects and Methods

The present study was performed using the baseline data from the Progredir Cohort Study. Briefly, patients from *Hospital das Clinicas* outpatient service, Sao Paulo, Brazil, a tertiary care facility, were invited to participate in the study. Inclusion criteria were: age ≥30 years-old and at least two measurements of serum creatinine (with a minimum interval of 3 months) ≥1.6mg/dL for men and ≥1.4mg/dL for women in a computer-screening including all ambulatory services. Exclusion criteria were: hospitalization in the last 6 months, acute myocardial infarction in the last 6 months, autoimmune diseases, pregnancy, psychiatric diseases, ongoing chemo or immunosuppressive therapy, ongoing RRT, glomerulonephritis, HIV/AIDS infection, C and B hepatitis, and any organ transplantation. Recruitment took place from March 2012 to December 2013, and 454 participants were enrolled. The study was approved by two local Ethics Committees and written informed consent was obtained from all participants.

Each participant visited the research center for interviews and clinical exams according to standard protocols. Interview and clinical examination were performed by trained personal under strict quality control. Participants were firstly submitted to anthropometric measures using light clothes. Blood pressure (BP) was measured using a validated oscillometric device (Omron HEM 705CPINT). Three measurements were performed at 1-minute intervals and the mean was calculated. Overnight fasting blood samples, 24hour and spot urine were collected. A standard 75-g oral glucose tolerance test was administered to all participants without known diabetes. Urine and blood aliquots were prepared and stored at -180°C in nitrogen.

Serum and urinary creatinine concentrations were determined by the Jaffé reaction and proteinuria was determined by colorimetric assay (pyrogallol). Glomerular filtration rate was estimated by the CKD-EPI [12] equation and 24h proteinuria was corrected for 1,73m^2^. Urinary and serum RBP assays were performed by the laboratory of the *Hospital do Rim e Hipertensão—Universidade Federal de São Paulo*. Urinary RBP was measured using an immunoenzymatic assay with monoclonal antibody and expressed as mg/g urinary creatinine, with a reference range of 0.10–2.7 mg/g creatinine [13]. Serum RBP was measured using a test System for turbidimetry with a polyclonal rabbit anti-human RBP antibody (DAKO/ Biogen), with a reference range of 26-60mg/L. Other laboratorial data were determined using conventional techniques. Albumin and creatinine were also performed in spot urine to calculate albumin-to-creatinine ratio (ACR). Normoalbuminuria was defined as an ACR ≤ 30μg/mg creatinine, microalbuminuria was defined as an ACR 30–300μg/mg creatinine and macroalbuminuria ≥ 300μg/mg creatinine.

Transthoracic echocardiography was performed using an Aplio XG; Toshiba Corporation, Tokyo, Japan, with a 2.5 MHz sector transducer. The readings consisted of qualitative analysis of echocardiographic findings and measurements of quantitative parameters. The carotid-femoral pulse wave velocity (PWV) was measured using a validated automated device (Complior, Artech Medicale, France) with the subject in the supine position. Intima media thickness (IMT) was measured in the outer wall of a pre-defined carotid segment of 1 cm in length from 1 cm below carotid bifurcation, during three cardiac cycles. We considered as valid acquired images that clearly visualized on both sides three reference points: (1) the anatomic guides for the common carotid arteries, (2) interfaces between the lumen and the vessel far wall and (3) interfaces between the media and the adventitia layers of the far vessel wall. We used MIA^™^ software to standardize the reading and interpretation of carotid scans.

For coronary artery calcium score, the participants underwent a non-contrast computed tomography. The scans were performed using a 64 detector computed tomography scanner (Philips Brilliance, Philips, Netherlands). After the scout images, each patient also underwent an ECG-gated prospective calcium score examination with a tube potential of 120 kV and a tube current adjusted to body habitus. Images were reconstructed in 2.5 mm slice thickness using standard filtered back projection. The CAC was expressed as Agatston units and the percentile was evaluated in a blinded fashion by an experienced cardiologist using semiautomatic software (Calcium Scoring, Philips Workstation). Patients with coronary stents were excluded from these analyses. CAC severity was further categorized as 0 or >0 and <100 or ≥100. [14]

### Statistical analysis

For descriptive analysis the variables were divided according to tertiles of urinary RBP. ANOVA and Kruskal—Wallis tests were performed for comparison of continuous variables, and the chi-square test was performed for comparison of categorical variables. For linear regression analyses, urinary RBP was log transformed. In our sample, CAC presented an asymmetric distribution instead of the characteristic zero-inflated distribution reported in non-CKD populations. Thus, generalized regression models on CAC using a gamma model were used to model this variable. Lastly, logistic regression models were built for the diagnosis of CKD-EPI eGFR <35 ml/min/1.73m^2^ and ROC curves of the probabilities of each model were compared (http://vassarstats.net/roc_comp.html). Statistical analyses were conducted using SPSS 17.0 software for Windows (SPSS, Inc.) and R software (calcification). All tests were two-tailed and a p value of 0.05 was considered significant.

## Results

Table 1 shows the baseline clinical and laboratorial variables for all participants according to tertiles of urinary RBP. Urinary RBP was inversely associated to renal function and positively associated to diabetes, female gender, systolic blood pressure, albuminuria, glycated hemoglobin, total-cholesterol, HDL-cholesterol, and PWV. Other parameters related to uremia such as anemia, acidosis and mineral metabolism were also associated to urinary RBP. Interestingly, urinary RBP was inversely associated with left atrium diameter and ejection fraction, as well as with self-reported history of acute myocardial infarction. No relationship between urinary RBP and carotid thickness was found. The relationship between urinary RBP and calcium score was assessed using gamma models. Although a positive association appeared in the univariate analysis, statistical sigificance was lost after adjustments for age, sex, eGFR and diabetes were made (not shown).

**Table 1:** Baseline clinical and laboratorial variables for all participants and according to urinary RBP tertiles.

	All		Tertile 1-uRBP		Tertile 2-uRBP		Tertile 3-uRBP		*p*
	n = 454		n = 144		n = 144		n = 144		
**Age (years; mean / std)**	67.5	11.9	67.5	12	67.8	11.3	66.5	12.4	0.64
**Sex (men; n / %)**	287	63.2%	108	75%	83	57.6%	84	58.3%	0.002
**Race (white; n/ %)**	300	66.1%	90	62.5%	97	67.8%	98	69.5%	0.42
**Hypertension (n/ %)**	409	90.1%	131	91%	125	87.4%	135	93.8%	0.18
**Diabetes (n/ %)**	208	45.8%	53	36.8%	72	50.3%	76	53.1%	0.04
**Acute myocardial infarction (n/ %)**	147	32.4%	47	32.9%	52	36.4%	44	30.8%	0.004
**Stroke (n/ %)**	73	16.1%	27	19.1%	30	21.4%	15	10.8%	0.09
**Systolic blood pressure (mmHg; mean / std)**	140	24.1	133.1	20.3	140.3	25.1	146.3	24.3	<0,0001
**Diastolic blood pressure (mmHg; mean / std)**	76.2	12.9	74.2	12.3	76.5	13.6	77.5	12.2	0.08
**Body-mass index (mean / std)**	29.4	5.4	30.3	6.5	29.1	4.6	29	5.1	0.11
**Potassium (mEq/L; mean / std)**	4.6	0.5	4.6	0.5	4.5	0.5	4.7	0.5	0.001
**Urea (mg/dL; median/ IQR)**	69	54–89	63	50–89	64	51–79	80	63,5–97	<0,0001
**Creatinine (mg/dL;median / IQR)**	1.7	1,4–2,1	1.6	1,3–1,9	1.5	1,3–1,8	2.1	1,7–2,6	<0,0001
**Albuminuria (mg/g creat; median / IQR)**	80	15–640	17	11–119	61	21–310	654	94–1759	<0,0001
**Urinary RBP (mg/g creat; median / IQR)**	0.29	0,08–1,47	0.05	0,03–0,08	0.29	0,18–0,42	3.09	1,47–10,69	<0,0001
**CKD-EPI eGFR (ml/min/1.73 m^2^ mean / std)**	38.4	14.6	42.5	15.7	43.0	13.6	29.5	10.7	<0,0001
**Phosphorus (mg/dL; mean / std)**	3.6	0.6	3.6	0.7	3.6	0.6	3.8	0.6	<0,0001
**Calcium (mg/dL; mean / std)**	9.6	0.6	9.5	0.6	9.6	0.5	9.5	0.6	0.16
**Parathormone (pg/mL; median / IQR)**	93	64–143	88	55–136	88	63,5–126	114	77–184	0.001
**Serum RBP (mg/L; median / IQR)**	66.1	55,2–79,8	63.3	51,1–77,6	60.5	53,9–78,3	75	62,1–85,7	<0,0001
**Glycemia (mg/dL;median / IQR)**	104	95–126	102	95,0–120,5	108.5	94,5–129,5	107.5	92,5–136	0.69
**Glycated hemoglobin (%; median / IQR)**	6.2	5,8–7,2	6.1	5,7–6,7	6.3	5,8–7,5	6.4	5,9–7,7	0.003
OGTT (mg/dl; mean / std)*	155	51.8	163	52.7	145.1	48.1	155.1	54.5	0.13
HOMA-IR (mediana/ IQ)*	3.4	2,3–5,8	4.2	2,4–6,5	2.9	2,2–5,0	2.8	2,2–5,3	0.02
**Insulinemia (μUI/mL; IQ)**	16.1	10,4–25,4	17.1	10,9–26,6	16.4	11,4–25,5	14.5	9,8–24,6	0.30
**Total cholesterol (mg/dL mean / std)**	168.6	39.9	161.9	35.3	169.8	42.6	173.6	41.7	0.04
**LDL-cholesterol (mg/dL; mean / std)**	91.4	32.2	89	28.4	90.3	35.4	93.9	33.1	0.42
**HDL-cholesterol (mg/dL; mean / std)**	46	14.3	42.9	10.8	47.2	17.6	47.7	13.7	0.007
**Triglycerides (mg/dL; median / IQR)**	142	99–192	143	101,5–188	141	99,5–204	141	94,0–196,5	0.89
**Serum pH (mean / std)**	7.4	0.039	7.4	0.040	7.4	0.037	7.3	0.038	<0,0001
**Bicarbonate (mmol/L; mean / std**	25.6	2.9	25.9	3.0	26.1	2.7	24.6	2.9	<0,0001
**Hemoglobin (g/dL; mean / std)**	13.1	1.9	13.5	2.0	13.2	1.7	12.7	1.9	0.003
**Albumin (mg/dL; mean / std)**	4.3	0.3	4.3	0.3	4.4	0.3	4.2	0.3	0.001
**Pulse wave velocity (cm/s; mean / std)**	12.8	3.0	12.1	3.1	12.5	2.8	13.6	2.8	<0,0001
**Left atrium diameter (mm; mean / std)**	41.4	5.4	42.5	5.6	41.7	6.2	40.2	4.1	0.001
**Ejection fraction (median / IQR)**	0.7	0,6–0,7	0.62	0,53–0,69	0.67	0,58–0,70	0.67	0,6–0,70	<0,0001
**Intima media thickness (mm; mean / std)**	0.75	0.20	0.77	0.24	0.73	0.19	0.73	0.18	0.13
**Agatston score (median / IQR)**	165	8–785	134	8–635	174	2–1002	166	2,5–786	0.76
**Agatston>100 (n/%)**	205	55.1%	63	53.8%	62	54.4%	69	55.6%	0.96

* calculated for participants without known diabetes

Next, we performed several multivariate models on urinary RBP (log transformed due to very skewed distribution), shown in Table 2. In the first models (Model 1), which were adjusted only for sex, age and CKD-EPI eGFR, most variables from Table 1 remained associated to urinary RBP. However, in Model 2, adjusted for the same variables than Model 1 plus diabetes and SBP, urinary RBP remained positively associated to albuminuria, serum phosphorus, HDL-cholesterol and PWV, and inversely associated to CKD-EPI eGFR, bicarbonate, serum albumin and left atrium diameter. In Model 3, all variables from Model 1 were used in a stepwise forward procedure and only CKD-EPI eGFR, albuminuria, left atrium diameter, SBP and bicarbonate remained significantly and independently associated to urinary RBP.

**Table 2:** Linear regression models on log urinary RBP.

	B	Std. Error	95%CI		*p*
*Model 1*: *adjusted for sex*, *age and CKD-EPI eGFR*					
**CKD-EPI eGFR (ml/min/1.73 m^2^)**	-.026	.003	-.031	-.021	<0,0001
**Diabetes**	.238	.076	.089	.387	.002
**Acute myocardial infarction**	-.063	.091	-.242	.116	.49
**SBP (mmHg)**	.009	.002	.006	.012	<0,0001
**Albuminuria (mg/g creat)**	.0002	.00003	.0001	.0003	<0,0001
**Glycated hemoglobin (%)**	.071	.027	.019	.124	.01
HOMA-IR*	-.025	.016	-.056	.006	.12
**Serum RBP (mg/L)**	.0001	.002	-.003	.003	.93
**Total cholesterol (mg/dL)**	.001	.001	-.001	.003	.42
**HDL-cholesterol (mg/dL)**	.007	.003	.001	.012	.01
**Potassium (mEq/L)**	.085	.078	-.067	.238	.27
**Phosphorus (mg/dL)**	.145	.064	.019	.272	.02
**Parathormone (pg/mL)**	-.0001	.0004	-.001	.001	.85
**Bicarbonate (mmol/L)**	-.037	.014	-.064	-.010	.01
**Hemoglobin (g/dL)**	-.020	.024	-.066	.027	.40
**Albumin (mg/dL)**	-.272	.127	-.521	-.022	.03
**Pulse wave velocity (cm/s)**	.053	.015	.024	.083	<0,0001
**Left atrium diameter (mm)**	-.015	.007	-.029	-.0001	.05
**Ejection fraction**	.535	.293	-.042	1.111	.07
*Model 2*: *adjusted for sex*, *age and CKD-EPI eGFR*, *diabetes and SBP*					
**CKD-EPI eGFR (ml/min/1.73 m^2^)**	-.024	.003	-.029	-.019	<0,0001
**Albuminuria (mg/g creat)**	.0001	.00003	.0001	.0002	<0,0001
**Phosphorus (mg/dL)**	.121	.062	-.002	.243	.05
**HDL-cholesterol (mg/dL)**	.008	.003	.002	.013	.01
**Bicarbonate (mmol/L)**	-.040	.013	-.066	-.014	.002
**Albumin (mg/dL)**	-.187	.122	-.428	.053	.13
**Pulse wave velocity (cm/s)**	.020	.016	-.012	.052	.23
**Left atrium diameter (mm)**	-.014	.007	-.028	-.0002	.05
*Model 3*: *all variables from model 1*, *stepwise forward*					
**CKD-EPI eGFR (ml/min/1.73 m^2^)**	-.020	.003	-.025	-.014	<0,0001
**Albuminuria (mg/g creat)**	.0002	.00003	.0001	.0002	<0,0001
**Left atrium diameter (mm)**	-.024	.007	-.038	-.010	.001
**SBP (mmHg)**	.006	.002	.003	.009	.001
**Bicarbonate (mmol/L)**	-.038	.013	-.064	-.012	.005

*calculated only for those without diabetes

We then performed stepwise linear regression on urinary RBP after stratification of participants according to albuminuria categories (normo, micro and macroalbuminuria). In all strata, CKD-EPI eGFR remained in the model, showing an inverse and significant association with urinary RBP (Table 3).

**Table 3:** Stepwise linear regression models on log urinary RBP stratified by albuminuria categories (normo, micro and macroalbuminuria) among 454 participants.

	B	Std. Error	95%CI		*p*
*Normoalbuminuria (n = 155)*					
**Sex (men)**	-.255	.108	-.468	-.042	.02
**Diabetes**	.306	.102	.104	.508	.003
**CKD-EPI eGFR (ml/min/1.73 m^2^)**	-.008	.004	-.015	-.001	.03
*Microalbuminuria (n = 136)*					
**CKD-EPI eGFR (ml/min/1.73 m^2^)**	-.023	.004	-.031	-.015	<0,0001
**SBP (mmHg)**	.006	.002	.001	.011	.01
*Macroalbuminuria (n = 150)*					
**CKD-EPI eGFR (ml/min/1.73 m^2^)**	-.020	.005	-.030	-.010	<0,0001

**Fig 1:**
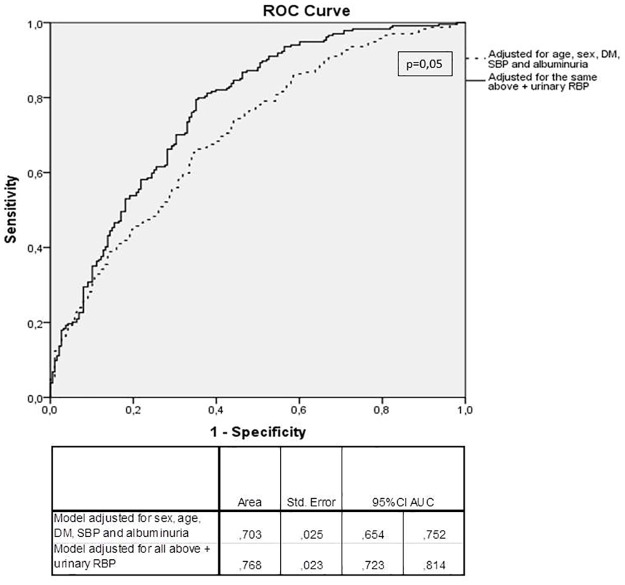
ROC curves for two models (with and without urinary RBP) for diagnosis of CKD-EPI eGFR<35ml/min/1.73m^2^ among 454 participants.

Lastly, we classified participants according to a CKD-EPI eGFR cut-off of 35 ml/min/1,73m^2^. We performed two logistic regression models for the diagnosis of a CKD-EPI eGFR <35 ml/min/1,73m^2^: one adjusting for sex, age, diabetes, SBP and albuminuria and the second adjusting for sex, age, diabetes, SBP, albuminuria and urinary RBP. Fig 1 shows that the model including urinary RBP had a better performance than the model without urinary RBP (p = 0,05 for non-parametric test comparing the two models).

## Discussion

Our results suggest that urinary RBP is significantly and inversely associated to renal function, even after adjustments for several confounding variables. Besides showing a consistent and independent relation to renal function in all analysis performed, urinary RBP added accuracy for predicting low eGFR in this cross-sectional analysis in comparison to a model adjusted for the most important and traditional risk factors for CKD, including albuminuria.

In the Progredir Study, glomerulonephritis and transplantation were not included and proximal tubulopathies account for a very low prevalence in general CKD. By showing that urinary RBP associates consistently with renal function in this scenario, our results indicate that urinary RBP may work as a biomarker of CKD in general, a performance beyond the three classical pathological conditions that have been previously shown to be associated with urinary RBP.

In addition, the association between eGFR and urinary RBP remained significant in all strata of albuminuria. This is particularly important in the setting of normoalbuminuric CKD, since this is a population in whom no laboratorial biomaker besides renal function itself is available. Albuminuria has been widely used as a surrogate marker of CKD. Nonetheless, it has been recognized that around 30% of CKD patients may present in fact a normoalbuminuric disease [15] and that renal function decline may precede evolution to macroalbuminuria and occurs in 10% of normoalbuminurics and 35% of microalbuminuria type 1 diabetic patients [16].

These results raise the question of why urinary RBP can be a marker of CKD in general. One hypothesis is that by being related to proximal tubular function, urinary RBP might reflect tubule-interstitial fibrosis, which is a well-recognized and powerful histologic predictor of CKD. A recent study showed that urinary RBP was correlated to the extent of interstitial fibrosis in kidney biopsies from 189 patients with various etiologies of CKD (glomerular, interstitial and vascular diseases) [17]. Unfortunately, in our study, we could not evaluate this hypothesis, since kidney biopsy is not routinely performed in CKD. Another possibility is that RBP appearance in urine indicates a failure in one of the mechanisms related to proximal tubule function. In physiological conditions, urinary RBP is expected to be <0,4mg/g creatinine. Higher levels may occur in three conditions: (1) due to an impairment in the glomerular filtration barrier, as occurs in nephrotic syndrome, (2) when there is massive production of low molecular weight protein, overcoming the capacity of proximal tubular reabsorption, as seen in cast nepropathy or (3) in conditions where a direct impairment in the reabsorption machinery is the phenotype, as occurs in Dent´s disease and cystinosis. All these mechanisms (particularly the first and third) might be operating in several etiologies of CKD, hinting on why urinary RBP can have an overall good performance in CKD in general.

Another interesting finding was the inverse association between urinary RBP and left atrium diameter. Curiously, this result was in the opposite direction of the relationship found for urinary RBP and SBP or PWV. Unfortunately, we could not further investigate the reasons for this. One hypothesis is that urinary RBP might discriminate to some extent pre-glomerular insults from intrinsic renal insults (glomerular, tubular) that predominate in diseases such as diabetic kidney disease, chronic interstitial nephritis, among others. In this sense, it would be interesting not only to evaluate if this inverse association between urinary RBP and left atrium diameter is confirmed in other CKD studies, but also test if urinary RBP can discriminate type 2 and type 4 cardio-renal syndromes [18–20].

Serum RBP and urinary RBP showed a positive association. It has been previously proposed that high levels of urinary RBP could be a consequence of high serum RBP levels [21,22]. RBP4, which accounts for almost the total content of circulating RBP, has been recently recognized as an adipocine, and is related to insulin resistance, obesity, diabetes and fat mass [23–26]. Thus, RBP4 could confound the relationship between urinary RBP and renal function or cardiovascular risk factors. In addition, decreasing eGFR is related to increasing serum RBP, a fact that could contribute to this confounding effect. Once adjustments were made, with a particular emphasis on renal function, the positive association between serum and urinary RBP was no longer significant. In addition, the other relationships described in the manuscript were also independent of the effect of serum RBP.

Our study has some important limitations. First, data is cross-sectional. To better understand how much information urinary RBP can add as a biomarker of CKD and CKD progression it would be important to test its performance in prospective studies and also in earlier stages of the disease. Progredir Cohort follow-up is ongoing and we will evaluate the role of urinary RBP in relation to clinical events in the near future. Second, as in other CKD cohorts, histological data is not available, since most patients with CKD (particularly considering the inclusion and exclusion criteria used in the Progredir Study) are not candidates to kidney biopsy. Thus, we could not test some hypothesis raised by our results. Third, we did not measure other tubular markers. It would be interesting to compare the performance of urinary RBP to those of other markers related to tubular function.

In conclusion, our results suggest that urinary RBP is significantly associated to renal function in general CKD and should be further explored as a predictive and independent marker of CKD progression.
